# Predicting *CD27* expression and clinical prognosis in serous ovarian cancer using CT-based radiomics

**DOI:** 10.1186/s13048-024-01456-7

**Published:** 2024-06-22

**Authors:** Chen Zhang, Heng Cui, Yi Li, Xiaohong Chang

**Affiliations:** Department of Obstetrics and Gynecology, Peking University People’s Hospital, No. 11 Xizhimen South Str., Xicheng District, Beijing, 100044 China

**Keywords:** Serous ovarian cancer, *CD27*, Radiomics model, Clinical outcome, Immune-cell infiltration

## Abstract

**Background:**

This study aimed to develop and evaluate radiomics models to predict *CD27* expression and clinical prognosis before surgery in patients with serous ovarian cancer (SOC).

**Methods:**

We used transcriptome sequencing data and contrast-enhanced computed tomography images of patients with SOC from The Cancer Genome Atlas (*n* = 339) and The Cancer Imaging Archive (*n* = 57) and evaluated the clinical significance and prognostic value of *CD27* expression. Radiomics features were selected to create a recursive feature elimination-logistic regression (RFE-LR) model and a least absolute shrinkage and selection operator logistic regression (LASSO-LR) model for *CD27* expression prediction.

**Results:**

*CD27* expression was upregulated in tumor samples, and a high expression level was determined to be an independent protective factor for survival. A set of three and six radiomics features were extracted to develop RFE-LR and LASSO-LR radiomics models, respectively. Both models demonstrated good calibration and clinical benefits, as determined by the receiver operating characteristic (ROC) curves, calibration curves, and decision curve analysis. The LASSO-LR model performed better than the RFE-LR model, owing to the area under the curve (AUC) values of the ROC curves (0.829 vs. 0.736). Furthermore, the AUC value of the radiomics score that predicted the overall survival of patients with SOC diagnosed after 60 months was 0.788 using the LASSO-LR model.

**Conclusion:**

The radiomics models we developed are promising noninvasive tools for predicting *CD27* expression status and SOC prognosis. The LASSO-LR model is highly recommended for evaluating the preoperative risk stratification for SOCs in clinical applications.

**Supplementary Information:**

The online version contains supplementary material available at 10.1186/s13048-024-01456-7.

## Introduction

Ovarian cancer (OC) is the second leading cause and deadliest type of gynecological malignancy worldwide, estimated to account for 19,710 new cases and 13,270 deaths in the US in 2023 [1]. This debilitating disease is highly heterogeneous and comprises multiple distinct histologic subtypes with different risk factors, origins, etiologies, molecular biology, and prognoses. Serous ovarian cancer (SOC) is the most common histological subtype with the least favorable prognosis [2]. Despite intense efforts and progress in therapeutic options over the past few decades, the overall survival (OS) for patients with OC is far from satisfactory [3]. Classic prognostic indicators such as clinicopathological characteristics, serum carbohydrate antigen 125 (CA125), ultrasound, and computed tomography (CT) can no longer meet the clinical needs of precision medicine. Therefore, urgent progress is required to explore novel prognostic markers that are beneficial for stratifying patients to provide new indicators for precision medicine.

The cluster of differentiation 27 gene (*CD27*, also known as TNFRSF7) is a member of the tumor necrosis factor receptor superfamily (TNFRSF) and frequently induces both costimulatory and apoptosis-inducing molecules to facilitate anti-tumor and anti-infection immunity [4–6]. *CD27* plays a crucial role in regulating B-cell activation and immunoglobulin synthesis [10, 11] by interacting with its only natural ligand *CD70* (*CD27*L), which is transiently expressed on antigen-activated immune cells [4, 7, 8]. This interaction transduces signals leading to the activation of pathways, including nuclear factor-kappa B and mitogen-activated protein kinase 8/c-jun N-terminal kinase [5, 9]. Moreover, *CD27* is expressed on tumor-infiltrating lymphocytes (TILs) and can transmit signals to T and NK cells across a variety of tumors [5]. Agonism of the costimulatory *CD27*-*CD70* pathway has been studied as a promising target for therapeutic intervention in various tumor types [10]. For instance, varilumab, a *CD27* agonizing monoclonal antibody, has been extensively studied as a monotherapy and checkpoint inhibitor therapy in several hematologic and solid tumor types, including Hodgkin’s lymphoma, non-Hodgkin’s lymphoma, and OC [10].

Images, such as CT images, may contain mineable information, reflecting the underlying pathophysiology of a tumoral tissue [11]. As an emerging translation field, radiomics investigates the association between quantitative high-dimensional data extracted from imaging examinations and clinical data to construct a prediction model, which eventually improves evidence-based personalized medical decision-making [12, 13]. In recent years, radiomics has shown great promise in predicting tumor diagnosis, molecular subtype, therapeutic effect, and survival in patients with various tumor types, including breast cancer [14, 15], lung cancer [16, 17], and OC [18–20]. A systematic review demonstrated that radiomics models have shown promising results as predictors of OS and progression-free survival (PFS) in patients with OC, but larger studies are needed to demonstrate clinical applicability [20]. However, no study has used radiomics features to predict the expression levels of *CD27* and evaluate its prognostic value in SOC.

In this study, we innovatively constructed radiomics models based on preoperative CT and clinical information for the noninvasive prediction of *CD27* expression and survival prognosis in patients with SOC. Using bioinformatics analysis, the underlying molecular mechanism of *CD27* and its possible association with the immune microenvironment were investigated.

## Methods

### Study cohort and data acquisition

**Fig. 1:**
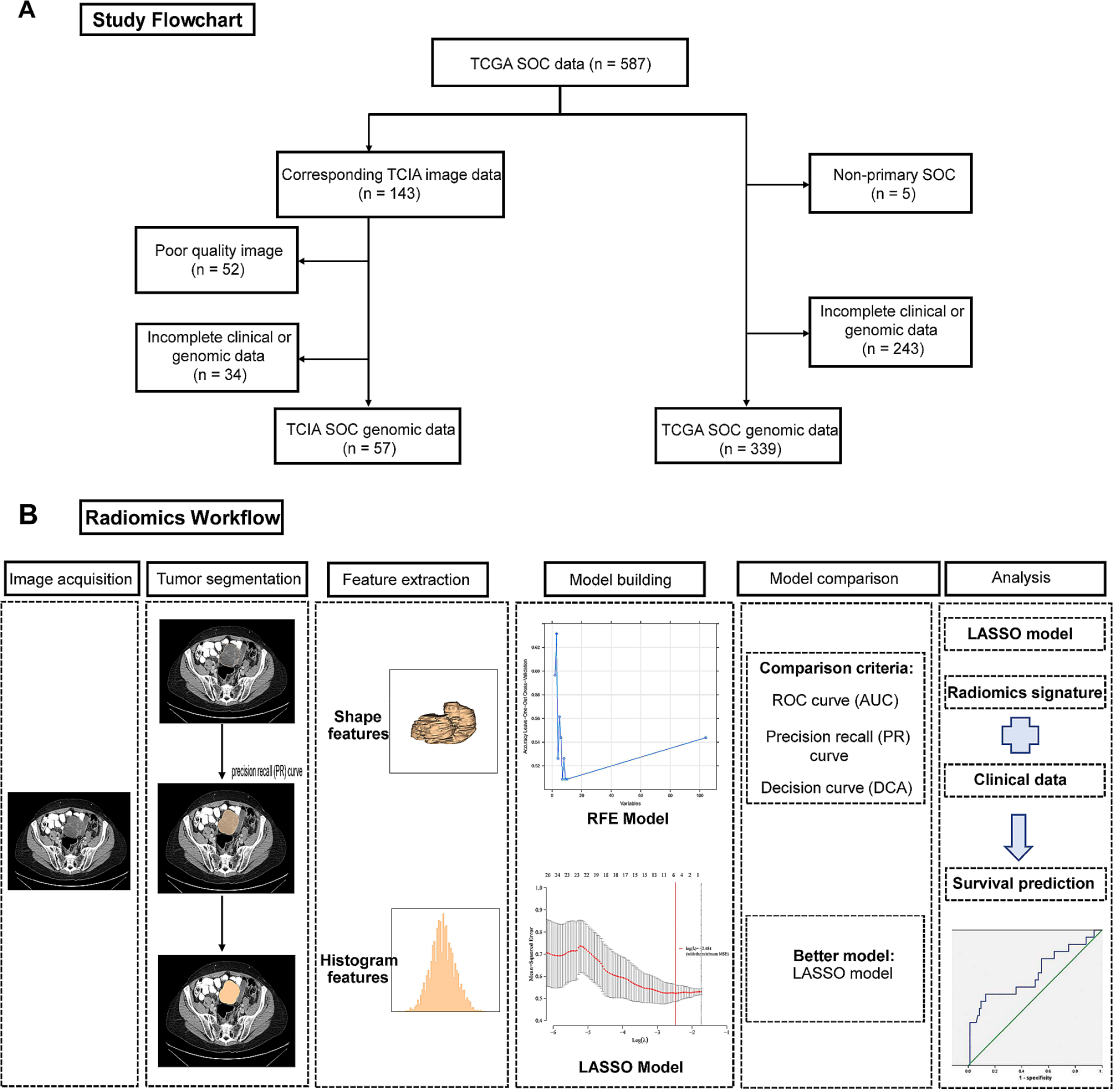
Flow chart in the study. **(A)** Gene and image data screening process. **(B)** The radiomics workflow. SOC: serous ovarian carcinoma; TCGA: The Cancer Genome Atlas; TCIA: The Cancer Imaging Archive; RFE, recursive feature elimination; LASSO: least absolute shrinkage and selection operator

To investigate the prognostic value of *CD27* expression and construct radiomics prediction models, this retrospective study used imaging (including clinical and follow-up data) and gene expression data (including clinical and follow-up data) from The Cancer Imaging Archive (TCIA, https://www.cancerimagingarchive.net/) and The Cancer Genome Atlas (TCGA, https://portal.gdc.cancer.Gov/), respectively. Three hundred thirty-nine patients with pathologically confirmed primary SOC from the TCGA dataset were enrolled in the study. Among these, 57 qualified cases had preoperative CT images stored in the TCIA. TCGA data were used to evaluate the prognostic value of *CD27* expression, and the images were used for feature extraction and radiomics model construction. The exclusion criteria included non-primary SOC cases, samples with incomplete clinical or genomic data, samples with poor-quality images, and images with no corresponding clinical or genomic data. A flowchart of this study is shown in Fig. 1.

### Identification of CD27 as a DEG in SOC

Based on the expression of *CD27* in the TCGA dataset, all subjects included in the study were dichotomized into CD27^high^ and CD27^low^ groups by the cutoff values calculated using the R package “survminer.” UCSC Xena (https://xenabrowser.net/datapages/) RNA-Seq data in the “transcripts per kilobase million” (TPM) format were processed uniformly using Toil (portable open-source workflow software) [21]. RNA-seq data from SOC in TCGA and normal samples from the GTEx databank were extracted. To compare the *CD27* levels between groups, the RNA-seq data in the TPM format was log2 transformed, and the R package “ggplot2” was used for visualization. The correlation between *CD27* expression and the clinical characteristics was analyzed using Spearman’s rank correlation coefficients.

Kaplan–Meier survival curve analysis illustrated changes in the survival rates among groups. The log-rank test was used to compare the statistical difference of survival rates in different groups. The R package “survival” was used for each variable, and the R package “survminer” was used for conclusion and visualization.

Univariate and multivariate Cox regression analyses were used to investigate the relationships between one or more study factors and survival outcomes. The R packages “survival” and “forestplot” were used in the aforementioned analyses.

A subgroup analysis was performed using univariate COX regression analysis to determine the role of *CD27* expression (CD27^high^ and CD27^low^ groups) in the prognosis of patients in different covariate subgroups. The R packages “cmprsk”, “survival” and “forestplot” were used in the subgroup analysis.

### Correlation analysis between CD27 expression and immune-cell infiltration and immune genes

The gene expression files of the SOC samples were uploaded to the CIBERSORTx database (https://cibersortx.stanford.edu/), and immune cell infiltration was measured for each sample. The correlation between *CD27* expression and immune cell infiltration or immune gene expression was calculated using Spearman’s rank correlation coefficient.

### GO and KEGG enrichment analysis

To further identify the differentially enriched pathways between the CD27^high^ and CD27^low^ groups, Gene Ontology (GO) and Kyoto Encyclopedia of Genes and Genomes (KEGG) functional enrichment analyses were employed. In this study, we used GO and KEGG analyses to visualize the top 10 and 20 significantly enriched pathways, respectively. The R package “clusterProfiler” was used for enrichment analyses and “org.Hs.eg.db” for ID conversion.

### Tumor segmentation and feature extraction

Fifty-seven qualified SOC cases with preoperative CT images from TCIA and bioinformatics data from TCGA were divided and labeled into high- and low- expression groups using the cutoff value (1.0959) of *CD27* expression calculated by the R package “survminer.” Volumes of interest were delineated along each tumor contour by one radiologist and repeated by another radiologist in 10 randomly selected patients. A set of 107 radiomics features was extracted using pyRadiomics and standardized. The intraclass correlation coefficient (ICC) was used to assess the inter-reader reproducibility for both image segmentation and radiomics feature extraction. Radiomics features with an ICC ≥ 0.75 were selected for further exploration.

### Construction and evaluation of RFE-LR model and LASSO-LR model

Before modeling, feature selection was conducted using recursive feature elimination (RFE) implemented by the R package “caret” and the repeat (1,000 times) least absolute shrinkage and selection operator (LASSO) method implemented by the R package “glmnet”, respectively, to find the optimal set of features for an accurate model. Using the glm function from R package “stats,” the radiomics features screened out by the RFE algorithm and LASSO algorithm were fitted using the logistic regression (LR) algorithm to establish a dichotomous model for predicting *CD27* expression.

The performance of the models was evaluated, and an internal 5-fold cross-validation was performed. Receiver operating characteristic (ROC) and precision recall (PR) curves were plotted to assess diagnostic performance. The calibration degree of the model was demonstrated using calibration curves and the Hosmer–Lemeshow goodness-of-fit testing method. The clinical usefulness of the model was evaluated using a decision curve analysis (DCA). This radiomics model can generate the prediction probability of gene expression levels (radiomics score, Rad_score). The R packages “pROC,” “measures,” “ResourceSelection” and “modEvA” were used in these analyses. Wilcoxon test was used to test the difference of Rad_score between CD27^high^ and CD27^low^ groups, implemented by the R package “ggplot2.”

### Comparison of RFE-LR model and LASSO-LR model

To determine which model was better, the Delong test was used to compare the area under the curve (AUC) values before and after validation. The PR curve is also an important reference. The LASSO-LR model performed better and was used for further analyses.

We incorporated the Rad_score in the LASSO-LR model into clinical data to obtain 57 SOC cases from the TCGA database with the corresponding Rad_score. A time-dependent ROC curve was constructed to demonstrate predictive capability at different time points. A time-based AUC for predicting patient survival at different time points after SOC diagnosis was also established.

## Results

### Patient characteristics

In total, 339 patients with SOC from the TCGA database were included in our study (Fig. 1). The cut-off expression level was 1.096, dichotomizing patients into CD27^high^ (*n* = 141) and CD27^low^ (*n* = 198) groups. The study cohort statistics of the groups are presented in Table 1. A significant difference in the distribution of residual tumor disease was noted between the CD27^high^ and CD27^low^ groups (*P* = 0.033). However, no significant differences were observed in variables such as age, FIGO stage, lymphatic invasion, venous invasion, histologic grade, or chemotherapy condition between the two groups (all *P* > 0.05).

**Table 1:** Patient characteristics

Variables	Total (*n* = 339)	Low (*n* = 198)	High (*n* = 141)	*P*	Statistic
Age (years), n (%)				0.344	0.894
~ 59	175 (51.62)	107 (54.04)	68 (48.23)		
60~	164 (48.38)	91 (45.96)	73 (51.77)		
FIGO stage, n (%)				0.444	0.586
I/II	19 (5.60)	9 (4.55)	10 (7.09)		
III/IV	320 (94.40)	189 (95.45)	131 (92.91)		
Lymphatic invasion, n (%)				0.446	1.614
No	40 (11.80)	27 (13.64)	13 (9.22)		
Yes	91 (26.84)	51 (25.76)	40 (28.37)		
Unknown	208 (61.36)	120 (60.60)	88 (62.41)		
Histologic grade, n (%)				0.852	0.035
G1/G2	41 (12.09)	25 (12.63)	16 (11.35)		
G3/G4/GX	298 (87.91)	173 (87.37)	125 (88.65)		
Tumor residual disease, n (%)				0.033	8.713
No macroscopic disease	58 (17.11)	35 (17.68)	23 (16.31)		
1 ~ 10 mm	162 (47.79)	103 (52.02)	59 (41.84)		
10 mm~	86 (25.37)	48 (24.24)	38 (26.95)		
Unknown	33 (9.73)	12 (6.06)	21 (14.89)		
Venous invasion, n (%)				0.632	0.917
No	32 (9.44)	20 (10.10)	12 (8.51)		
Yes	59 (17.40)	37 (18.69)	22 (15.60)		
Unknown	248 (73.16)	141 (71.21)	107 (75.89)		
Chemotherapy, n (%)				0.206	1.598
No	21 (6.19)	9 (4.55)	12 (8.51)		
Yes	318 (93.81)	189 (95.45)	129 (91.49)		

### Clinical significance of CD27 in SOC

**Fig. 2:**
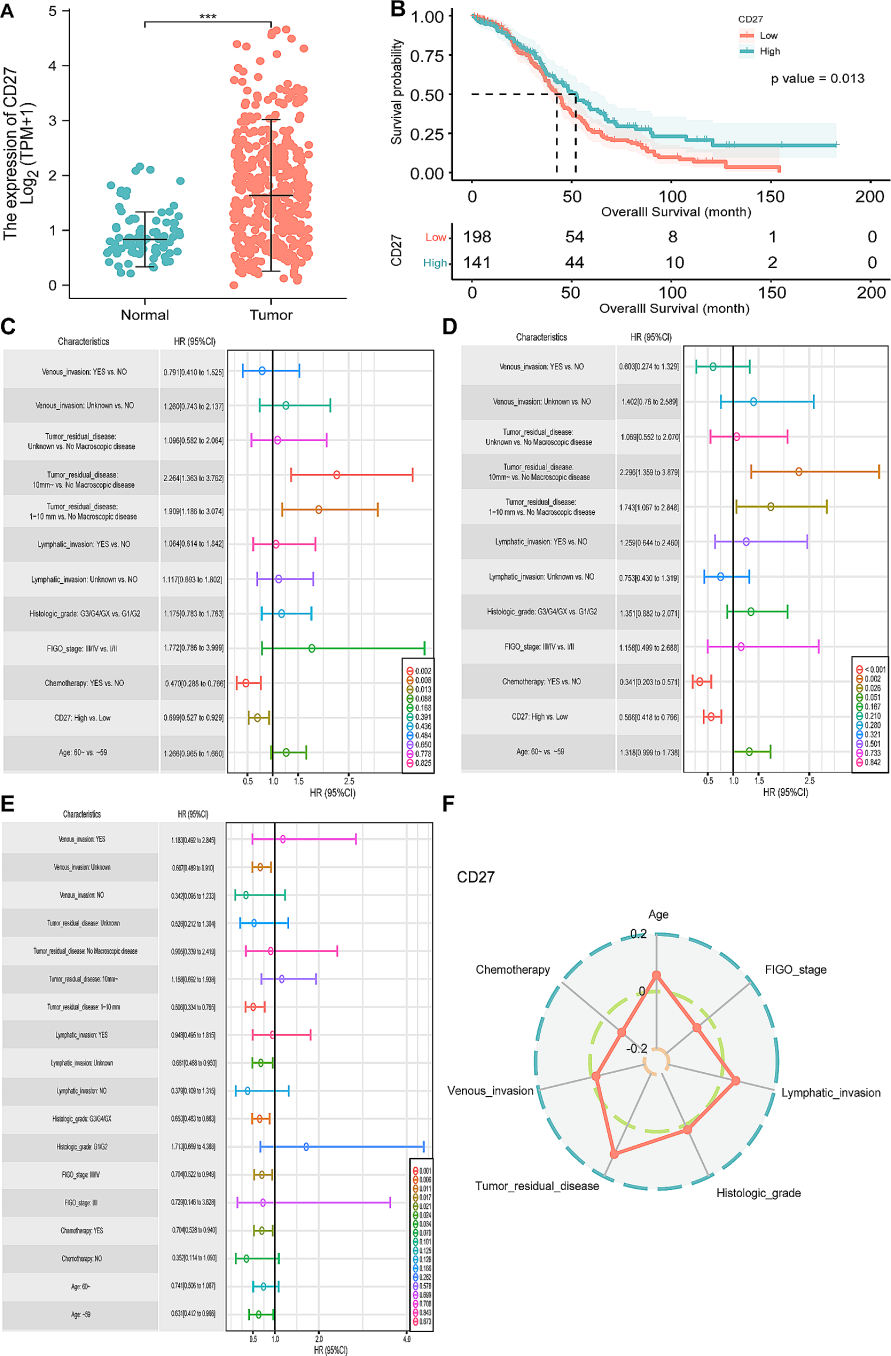
CD27 expression in SOC and its association with clinical characteristics. **(A)** CD27 expression was upregulated in SOC tissues than in normal tissues (^∗∗∗^*P* < 0.001). **(B)** Kaplan–Meier analysis showed that low CD27 expression was associated with a poor OS of patients with SOC (*P* = 0.013). **(C)** Univariate Cox regression analyses. **(D)** Multivariate Cox regression analyses. **(E)** Subgroup analyses. **(F)** Correlation analysis between CD27 expression level and clinical characteristics of SOC. SOC, serous ovarian carcinoma; OS, overall survival

As shown in Fig. 2A, *CD27* expression levels were significantly higher in SOC samples from the TCGA project than in normal ovary samples from the GTEx databank (*P* < 0.001). The median survival times in the CD27^low^ and CD27^high^ groups were 42.6 (95% confidence interval [CI], 38.6–46.3) and 52.1 (95% CI, 41.6–64.4) months, respectively. Kaplan–Meier survival analysis showed that a high expression level of *CD27* was associated with a better prognosis in terms of OS than a low expression level (*P* = 0.013, Fig. 2B). The results of the univariate Cox regression analysis indicated that variables, including high expression levels of *CD27* (hazard ratio [HR] = 0.699, 95% CI:0.527–0.929, *P* = 0.013) and chemotherapy (HR = 0.47, 95% CI:0.288–0.766, *P* = 0.002), were protective factors for SOC. However, Tumor_residual_disease: 1–10 mm (HR = 1.909, 95% CI:1.186–3.074, *P* = 0.008) or Tumor_residual_disease:10 mm (HR = 2.264, 95% CI:1.363–3.762, *P* = 0.002) correlated with poorer survival than No Macroscopic disease (Fig. 2C). After adjustment for other covariates included in the multivariate COX regression analysis, a high expression level of *CD27* (HR = 0.566, 95% CI:0.418–0.766, *P* < 0.001) and chemotherapy (HR = 0.341, 95% CI:0.203–0.571, *P* < 0.001) were independent protective factors for survival, and Tumor_residual_disease: 1–10 mm (HR = 1.743, 95% CI:1.067–2.848, *P* = 0.026) or Tumor_residual_disease: 10 mm~ (HR = 2.296, 95% CI:1.359–3.879, *P* = 0.002) were independent risk factors (Fig. 2D). In the univariate subgroup analysis, a high *CD27* expression level was protective in patients with SOC with the characteristics: aged ≤ 50 years (HR = 0.631, 95% CI:0.412–0.966, *P* = 0.034), FIGO_stage: III/IV (HR = 0.704, 95% CI:0.528–0.94, *P* = 0.017), Histologic_grade: G3/G4/GX (HR = 0.653, 95% CI:0.483–0.883, *P* = 0.006), Tumor_residual_disease: 1–10 mm (HR = 0.506, 95% CI:0.334–0.765, *P* = 0.001) and Chemotherapy: YES (HR = 0.704, 95% CI:0.522–0.949, *P* = 0.021) (Fig. 2E). Moreover, *CD27* expression levels were significantly associated with Tumor_residual_disease (*P* = 0.036; Fig. 2F).

### CD27 expression correlates with immune-cell infiltration and immune genes in SOC

**Fig. 3:**
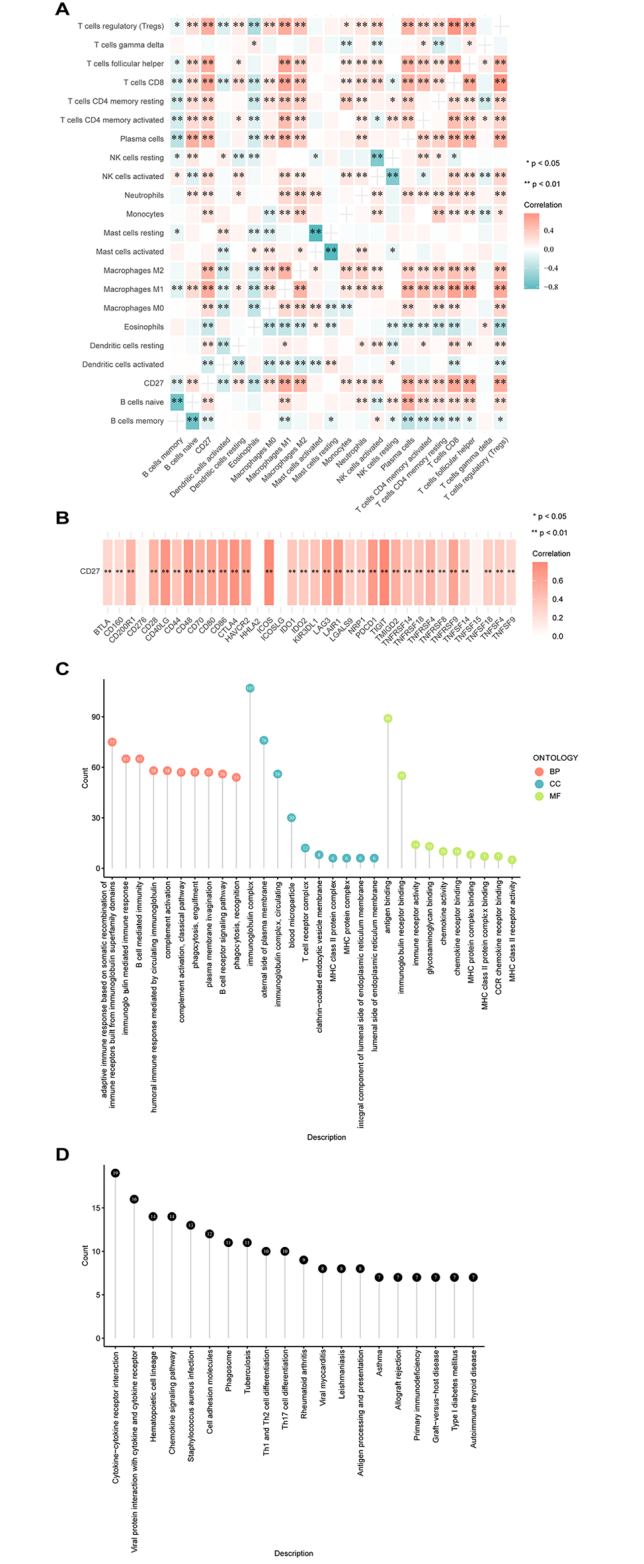
Correlation of the immune-cell infiltration, DEGs, and CD27 expression in SOC. **(A)** Correlation heat map between CD27 expression and immune-cell infiltration in SOC in CIBERSORTx database. **(B)** Correlation between CD27 expression and immune-related genes. **(C)** GO enrichment analysis of the DEGs related to CD27. **(D)** KEGG enrichment analysis of the DEGs related to CD27. SOC, serous ovarian carcinoma; GO, gene ontology; DEGs, differentially expressed genes; KEGG, Kyoto Encyclopedia of Genes and Genomes

Correlation heat maps revealed that *CD27* positively correlated with macrophages (M0, M1, and M2) and T cells, including T cells CD8, T cells follicular helper, regulatory T cells (Tregs), CD4 memory testing, and T cells CD4 memory activated, while negatively correlated with eosinophils and dendritic cell activation (Fig. 3A, all *P* < 0.01). Furthermore, *CD27* expression had a significant positive correlation with the expression levels of some immune-related genes, including *CTLA4*, *PDCD1*, *CD70*, *CD86*, *CD48*, *CD28*, and *CD80* (Fig. 3B, all *P* < 0.01).

### GO and KEGG enrichment analysis of DEGs associated with CD27 in SOC

GO enrichment analysis indicated that differentially expressed genes (DEGs) in the CD27^high^ and CD27^low^ groups were significantly enriched in pathways, including complement activation, T cell receptor complex, and immunoglobulin receptor binding (Fig. 3C). KEGG enrichment analysis revealed that the DEGs were significantly enriched in cytokine-cytokine receptor interactions (Fig. 3D).

### Construction and evaluation of RFE-LR model

**Fig. 4:**
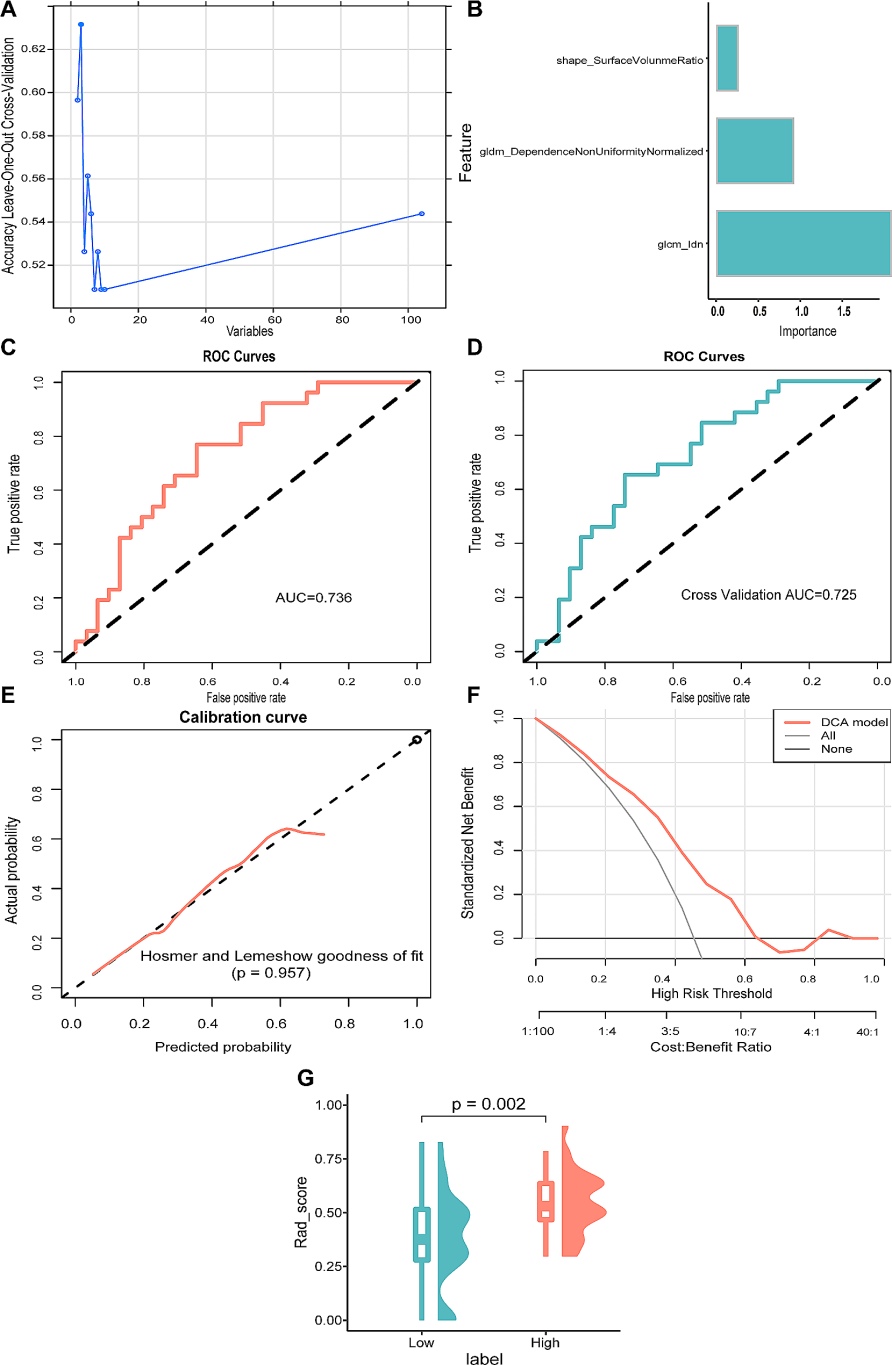
Construction and evaluation of the RFE-LR model. **(A)** Radiomics features with statistical differences using the RFE algorithm. **(B)** Importance of the selected features in the RFE-LR model. **(C)** ROC curve analysis of the RFE-LR model. **(D)** Cross-validation ROC curve analysis of the RFE-LR model. **(E)** Calibration-curve analysis of the RFE-LR model. **(F)** Hosmer–Lemeshow goodness-of-fit testing. **(G)** Prediction of CD27 expression level using the RFE-LR model. RFE, recursive feature elimination; LR, logistic regression; ROC, receiver operating characteristic curve

In total, 57 patients with SOC from the TCIA database were included in this study. The median ICC value of the image radiomics features was 0.975, and 104 out of 107 features (97.2% of the total features) with ICC ≥ 0.75 were enrolled for further analysis. After feature reduction using the RFE algorithm (Fig. 4A), three features remained for model construction. Their importance is shown in Table 2; Fig. 4B. The RFE-LR model showed favorable predictive ability, as shown by the ROC curve. This model produced an AUC of 0.736 (Fig. 4C) and 0.725 after internal 5-fold cross-validation (Fig. 4D); the PR-AUC of this model was 0.642 (Supplementary Fig. 1). Calibration curves and Hosmer–Lemeshow goodness-of-fit testing indicated that our prediction fits well with the actual *CD27* expression levels (*P* = 0.957, Fig. 4E). DCA analysis showed the high clinical practicality of the model (Fig. 4F). The AUC values before and after cross-validation showed no statistically significant differences (*P* = 0.867). Moreover, the Rad_score was significantly higher in the CD27^high^ group than in the CD27^low^ group (*P* < 0.01; Fig. 4G).

**Table 2:** Radiomics features by RFE-LR model

Item	Overall Impotence
original_shape_SurfaceVolumeRatio	0.219705626
original_gldm_DependenceNonUniformityNormalized	0.811868018
original_glcm_Idn	1.850713027

RFE, recursive feature elimination; LR, logistic regression.

### Construction and evaluation of LASSO-LR model

**Fig. 5:**
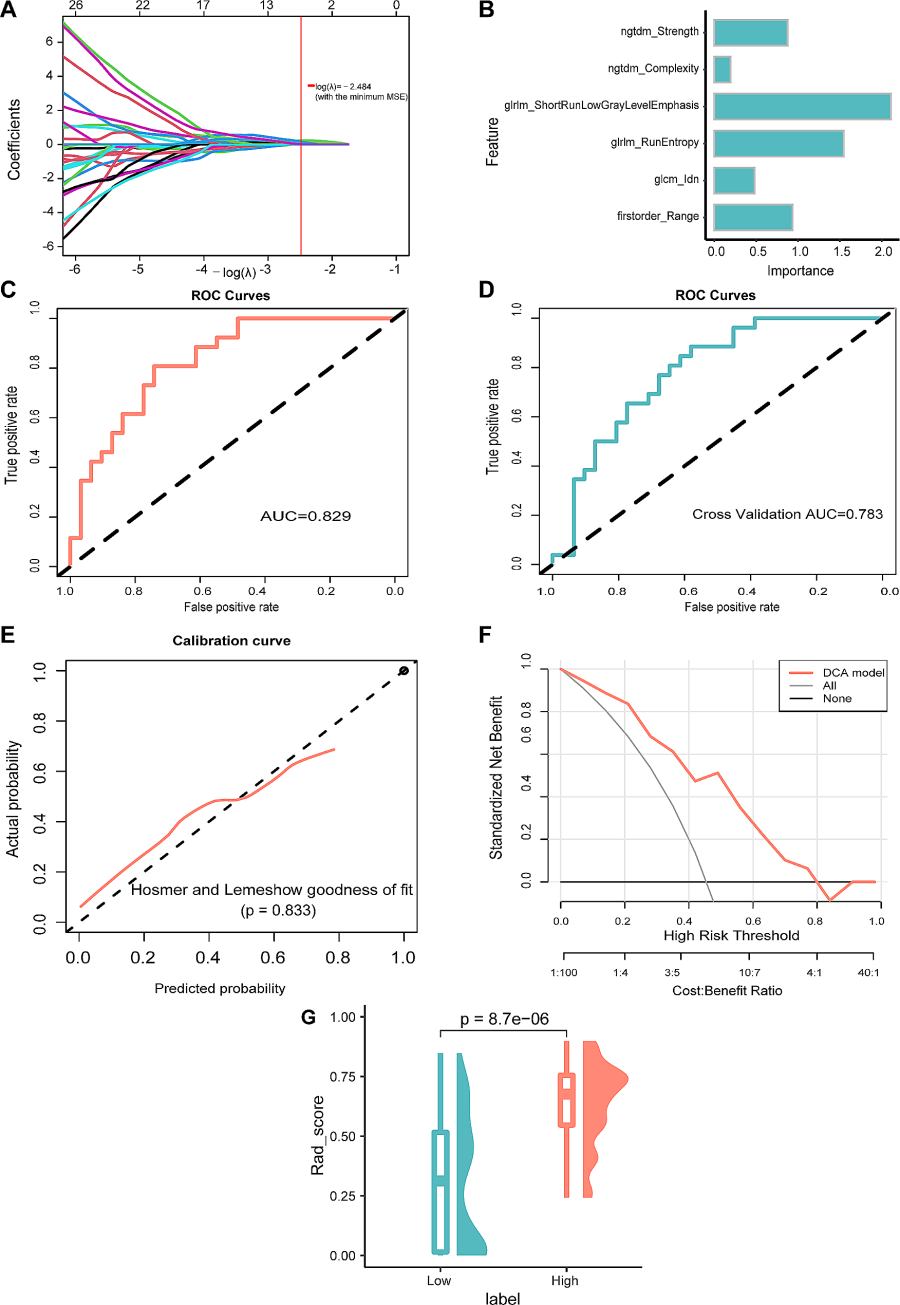
Construction and evaluation of LASSO-LR model. **(A)** Radiomics features with statistical differences using repeat LASSO algorithm. **(B)** Importance of the selected features in the LASSO-LR model. **(C)** ROC curve analysis of the LASSO-LR model. **(D)** Cross-validation ROC curve analysis of the LASSO model. **(E)** Calibration-curve analysis of the LASSO-LR model. **(F)** Hosmer–Lemeshow goodness-of-fit testing. **(G)** Prediction of CD27 expression level using the LASSO-LR model. LASSO, least absolute shrinkage and selection operator; LR, logistic regression; ROC, receiver operating characteristic curve

Six radiomics features were selected for inclusion in the LASSO-LR model (Fig. 5A). Figure 5B; Table 3 show the importance of the selected radiomics features. The LASSO-LR model showed a favorable predictive capacity, with an AUC of 0.829 (Fig. 5C) and an AUC after internal 5-fold cross-validation of 0.783 (Fig. 5D). The PR-AUC of this model was 0.780 (Supplementary Fig. 2). According to the calibration curves and Hosmer-Lemeshow goodness-of-fit testing, the LASSO-LR model revealed high conformity in predicting *CD27* expression levels compared with the actual value (*P* = 0.833, Fig. 5E). DCA analysis displayed preferable clinical practicality for the model (Fig. 5F). No statistical difference was observed in the AUC values before and after cross-validation (*P* = 0.566). The Rad_score distribution was remarkably different in the CD27^high^ and CD27^low^ groups, with a higher Rad_score in the CD27^high^ group (*P* < 0.001; Fig. 5G).

**Table 3:** Radiomics features by LASSO-LR model

Item	Overall Impotence
original_firstorder_Range	0.93416763
original_glcm_Idn	0.480737144
original_glrlm_RunEntropy	1.54478347
original_glrlm_ShortRunLowGrayLevelEmphasis	2.108777764
original_ngtdm_Complexity	0.193237187
original_ngtdm_Strength	0.874654247

LASSO, least absolute shrinkage and selection operator; LR, logistic regression.

### Model selection and survival analysis

**Fig. 6:**
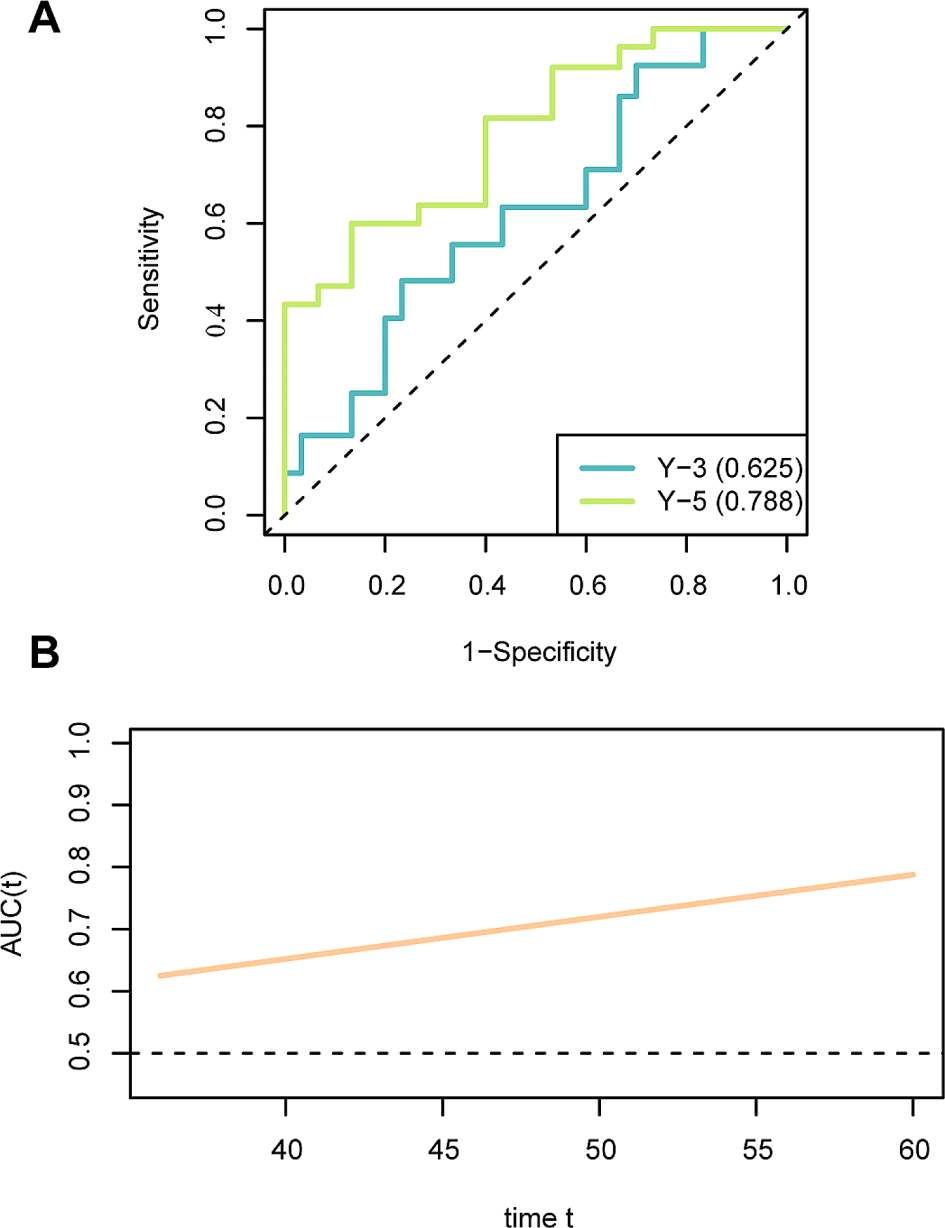
Prediction of OS in SOC using LASSO-LR model. **(A)** Time-dependent ROC curve constructed using the AUC value of the main variable at each time point (36 and 60 months). **(B)** The AUC value at different time points. OS, overall survival; SOC, serous ovarian carcinoma; LASSO, least absolute shrinkage and selection operator; LR, logistic regression; ROC, receiver operating characteristic curve; AUC, area under the curve

We compared the AUC values before and after validation in the RFE-LR and LASSO-LR models, respectively, using the Delong test, and no statistical difference was observed (*P* > 0.05). Based on the ROC (AUC:0.736 vs. 0.829; cross-validation AUC:0.725 vs. 0.783) and PR curves (AUC:0.642 vs. 0.780) of the two models, we found that the LASSO-LR model was superior to the RFE-LR model. Therefore, the LASSO-LR model was used for subsequent prognostic analyses. The results of time-dependent ROC curve analysis indicated that the AUC values of the Rad_score predicting OS at 36 months and 60 months were 0.625 and 0.788, respectively (Fig. 6A). The AUC increased over time (Fig. 6B).

## Discussion

The gold standard treatment for patients with epithelial ovarian cancer (EOC) comprises complete cytoreductive surgery, followed by platinum-based adjuvant chemotherapy with or without maintenance therapy [22]. A residual tumor of less than 1 cm is considered optimal after debulking surgery, and the amount of residual tumor tissue remains the strongest prognostic factor for survival [23]. Preoperative assessment of the scope and timing of primary surgery is a key issue. However, conventional CT techniques are insufficient for accurate and individualized imaging, and imaging analysis combined with biomarkers is currently a new hotspot. Our study developed CT-based radiomics models to preoperatively and noninvasively predict *CD27* expression levels and prognosis in patients with SOC, which is beneficial for personalized clinical decision-making.

However, few studies have investigated the role of *CD27* in OC. Swiderska et al. found that *CD27* could be a potential biomarker for the diagnosis of OC and is an unfavorable prognostic factor for OC [24]. CD27 + TILs were associated with improved prognosis in high-grade SOC [25]. Guo et al. observed that higher *CD27* expression indicated greater sensitivity to cisplatin treatment [26]. As the ligand for *CD27*, *CD70* expression can be induced by the platinum treatment of OC cells [27]. These results suggest that antibody-drug or antibody-drug conjugates in the *CD27*-*CD70* pathway may be promising immunotherapeutic regimens for patients with cisplatin-resistant OC [26, 27]. *CD27* is expressed on TILs [5], and a less differentiated TIL phenotype (CD27^+^TIL) is associated with favorable survival after incomplete cytoreductive surgery [25]. Varlilumab may have potential therapeutic implications in the treatment of OC [10, 28]. In a Phase I/II dose-escalation and cohort expansion study (NCT02335918), the combination of anti-PD1/PD-L1 antibody (nivolumab) and varlilumab resulted in 5 of 49 (10%) patients with OC achieving partial remission (PR), and 19 of 49 (39%) achieving stable disease (SD) [28]. Additionally improved clinical outcomes in a subset of patients, particularly patients with OC, have been associated with increased tumor expression of PD-L1 and CD8 + TILs [28]. Herein, *CD27* was identified as a differentially expressed prognosis-related gene in SOC, and its expression was associated with immune cell infiltration and immune genes, such as *CTLA4*, *PDCD1*, and *CD70*. Consequently, *CD27* signaling pathway targeting strategies may provide new insights into SOC.

The success of radiomics has been reported in its application in evaluating of ovarian masses [29], categorizing cancer subtypes [19, 30–32], predicting metastasis [33–35], recurrence [36], and survival [20, 29] in OC. Notably, radiomics models could predict the expression of targets and clinical outcomes in OC [18, 37–39]. Combining clinical and radiomics models may improve model performance when predicting BRCA mutations and PFS in OC [18]. Habitat radiomics using positron emission tomography/CT imaging can accurately predict Ki-67 status and stratify the prognosis of patients with OC [37]. Gao et al. showed that radiomics signatures from CT images can differentiate between the PD-1 expression status and OS in patients with OC [38]. A recent study revealed that a CT-based radiomics model using LASSO regression analysis could predict C-C motif chemokine receptor type 5 (CCR5) expression and survival in OC [39].

Targeting *CD27* in OC treatment has a potential application. However, the detection method of *CD27* is limited. The study constructed radiomics models to predict *CD27* expression in OC, aiming to provide a non-invasive method for detecting *CD27* and a tool for dynamic monitoring of molecular expression, thereby providing a useful strategy for precision medicine. Herein, the RFE-LR and LASSO-LR models based on *CD27* expression, radiomics, and clinical features were developed, and the performance of the two models were compared. Both models showed the capability and applicability of noninvasively predicting the expression of *CD27* in SOC. Furthermore, the LASSO-LR model performed better than the RFE-LR model in predicting the prognosis. In line with the literature [18, 37–39], our study demonstrates that combining biomarker-based features with standard radiomics offers a convenient and feasible strategy for improving prognosis prediction.

This study has some limitations. A limitation is its retrospective nature, so the findings require further validation. Another limitation is that all images were downloaded from a public dataset, and the sample size was relatively small. Hence, a multicenter study with good reproducibility should be conducted using independent cohorts.

## Conclusion

The expression levels of CD27 significantly influenced the clinical prognosis of patients with SOC. Radiomics models based on CT signatures and clinical data can preoperatively discriminate *CD27* expression levels and predict the prognosis of patients with SOC, providing a practical and noninvasive tool for predicting survival prognosis in patients with SOC.

### Electronic supplementary material

Below is the link to the electronic supplementary material.

Supplementary Material 1
