# Supplementary material for: Predicting CD27 expression and clinical prognosis in serous ovarian cancer using CT-based radiomics
Source: J Ovarian Res. 2024 Jun 22;17:131. doi: 10.1186/s13048-024-01456-7 (PMC11193901; doi:10.1186/s13048-024-01456-7)
Supplement: Supplementary file 1 — Supplementary Material 1 [file 13048_2024_1456_MOESM1_ESM.docx]

**
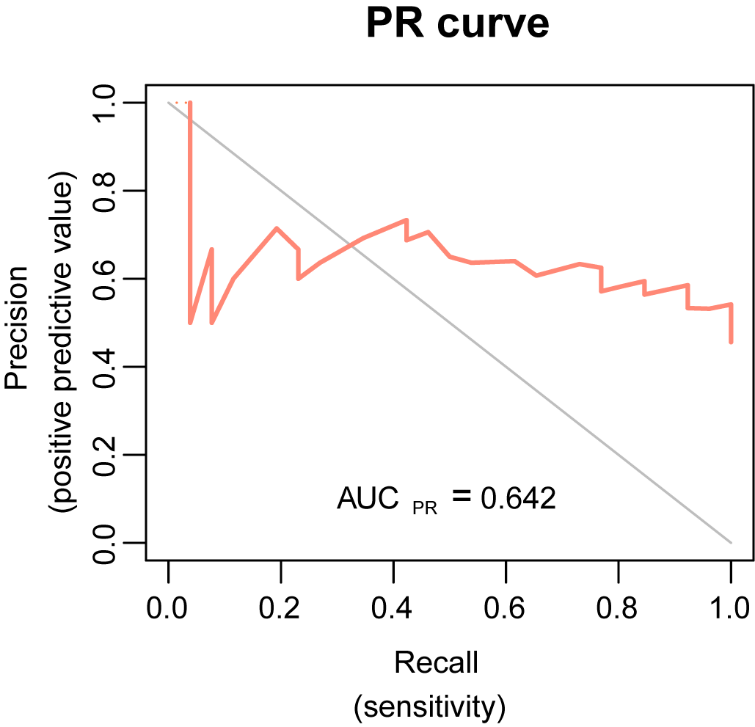
Supplementary Figure 1**. PR curve of the RFE-LR model.

Precision-recall, PR; RFE, recursive feature elimination; LR, logistic regression; AUC, area under curve.


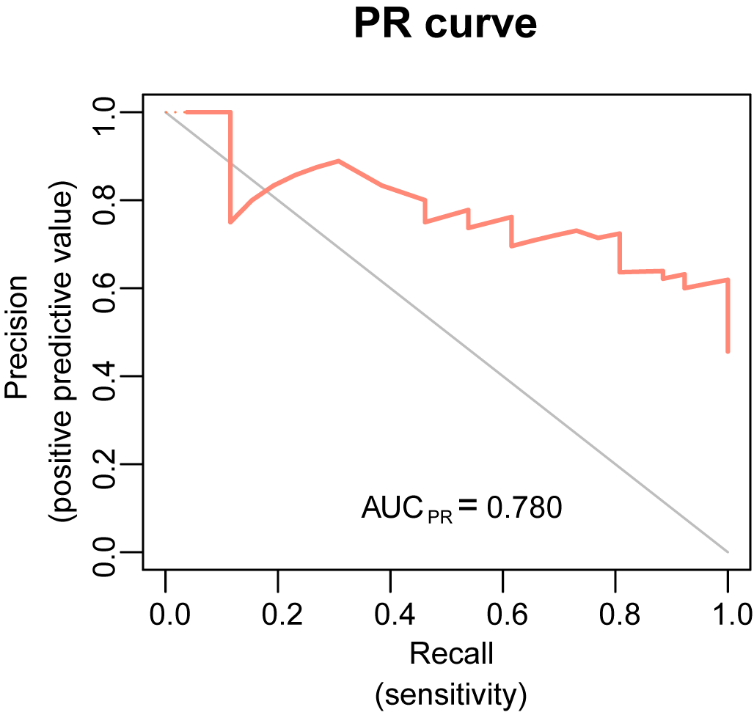
**Supplementary Figure 2**. PR curve of the LASSO-LR model.

Precision-recall, PR; LASSO, least absolute shrinkage and selection operator; LR, logistic regression; AUC, area under curve.
